# Determinants of medication adherence among older adults with type 2 diabetes using the health action process approach: A cross-sectional study

**DOI:** 10.1371/journal.pone.0332235

**Published:** 2025-09-25

**Authors:** Shiva Mohammadi, Bahareh khorami, Maryam Seyedtabib, Zhila Najafpour

**Affiliations:** 1 Department of Health care Management, School of Public Health, Ahvaz Jundishapur University of Medical Sciences, Ahvaz, Iran; 2 Department of Biostatistics and Epidemiology, School of Public Health, Ahvaz Jundishapur University of Medical Sciences, Ahvaz, Iran; UCSI University, MALAYSIA

## Abstract

**Background and aims:**

Medication adherence is a determinant of managing chronic disease. Failure to adhere to treatment can result in disease progression, increased hospitalizations, and a higher risk of complications and mortality. This study aimed to determine the level of medication adherence in older adults with type 2 diabetes based on the Health Action Process Approach (HAPA).

**Methods:**

This study is a descriptive-analytical cross-sectional study that was conducted on 179 older adults with type 2 diabetes. Data were collected using the Morisky Medication Adherence Scale (MMAS-8-Item) and the HAPA questionnaire. We used the chi-square test to compare adherence to medication by demographic characteristics and multiple binary logistic regression analysis to predict factors related to medication adherence based on the HAPA dimensions.

**Results:**

A total of 179 participants (87 men and 92 women) with a mean age of 64.65 ± 4.99 years were enrolled. Low medication adherence was reported by 62% of participants. No significant associations were found between socio-demographic factors (gender, marital status, education, employment, and income) and adherence levels. Logistic regression analysis revealed that smoking (OR = 4.309, 95% CI [1.18, 15.67], p = 0.027) and perceived barriers to adherence (OR = 1.036, 95% CI [1.01, 1.06], p = 0.001) were significantly associated with increased odds of medication non-adherence. Conversely, higher recovery self-efficacy (OR = 0.924, 95% CI: 0.86–0.99, p = 0.027) and coping planning (OR = 0.963, 95% CI: 0.93–0.99, p = 0.022) were associated with reduced odds of non-adherence. The most common self-reported reasons for suboptimal adherence were lack of affordability (17.5%), lack of family support (10%), and poor understanding of the disease (9.4%).

**Conclusion:**

This study highlights that older people had suboptimal adherence to medication. Smoking and perceived barriers were significant risk factors, increasing the likelihood of poor adherence. Conversely, higher levels of recovery self-efficacy and coping planning served as protective factors, reducing the risk of non-adherence. Policymakers and planners should consider the mentioned factors in designing interventions to change behavior for chronic diseases like diabetes.

## Introduction

Diabetes mellitus (DM) is becoming a serious public health problem with considerable social and economic burden worldwide. DM is notably prevalent among middle-aged and older adults [1]. Monitoring and controlling blood glucose levels is the ultimate goal to prevent early complications of diabetes, which requires long-term follow-up with uninterrupted access and adherence to medications [2,3].

Despite the availability of effective treatments, non-adherence to diabetes medication remains a major challenge, particularly among the older adults [4]. Evidence reports that nearly half of individuals with diabetes show poor adherence [1], with rates ranging from 36% to 93% for oral hypoglycemic agents and about 63% for insulin [5]. Such non-adherence increases the risk of complications, hospitalization, all-cause mortality, and imposes a substantial financial burden on both patients and health systems [5,6]. Importantly, adherence is a multifaceted behavior influenced by several factors [7]. Reported barriers include complex treatment regimens, involvement of multiple prescribers, ineffective patient–provider communication, lower socioeconomic status, and high out-of-pocket costs [8,9]. These findings highlight the need to identify modifiable determinants of adherence in order to develop targeted and effective interventions.

Several theoretical models have been applied to explain barriers to medication adherence, including the Health Belief Model, Social Cognitive Theory, the Theory of Planned Behavior, Self-Efficacy Theory, and Self-Regulation Theory [10]. Among these, the Health Action Process Approach (HAPA) has been particularly influential in understanding health behavior change. HAPA distinguishes between two interrelated phases: (1) the motivational phase, in which risk perceptions, outcome expectancies, and task self-efficacy contribute to the formation of behavioral intentions; and (2) the volitional phase, in which maintenance self-efficacy, recovery self-efficacy, action planning, and coping planning facilitate the translation of intentions into sustained health behaviors [11–13]. By addressing the well-documented gap between intentions and actual behavior, HAPA provides a comprehensive framework for studying adherence. Empirical research has supported the model, demonstrating that behavioral intention, task self-efficacy, coping planning, and coping self-efficacy are significant determinants of medication adherence across a range of chronic conditions, including diabetes, cardiovascular disease, and other long-term illnesses [14–17].

In Iran, the prevalence of high blood glucose is approximately 10% [18], which exceeds the estimated global prevalence. National reports further indicate that only 13% of individuals with diabetes in Iran effectively manage their hyperglycemia [19]. This inadequate control may be associated with nonadherence to prescribed medications among older adults diagnosed with type 2 diabetes. Therefore, the purpose of this study was to investigate the association between HAPA constructs and medication adherence among older adults with diabetes.

## Materials and methods

This cross-sectional study was conducted among patients with type 2 diabetes attending outpatient centers from 9-April to 20-July 2023 in Ahwaz, southwest of Iran.

### Participants and setting

Our participants included diabetes patients older than 60 years who had been taking diabetes medications for longer than six months. The individuals with type 2 diabetes included in this study were recruited from the two diabetes clinics affiliated with two tertiary hospitals. Patients were selected randomly with the help of a computer-generated list from the hospital database. We calculated the sample size using the prevalence-based formula n = z^2^ p (1-p)/ d^2^, where n is the sample size, (P) is the expected prevalence of non-adherence to medication (0.65) [20], (Z) is the statistic corresponding to the level of confidence (1.96), and (d) is precision (0.07). Based on information mentioned above, we obtained a sample size of 179. The inclusion criteria were: patients with type 2 diabetes under therapy with any type of medication for more than 6 months, able to speak Persian or Arabic, consenting to participate in the study. We excluded patients suffering acute severe medical illness, those with severe communication difficulties (dysphasia, severe hearing impairment, and so on), and those with a history of mental illness from the study.

### Data collection

Data were gathered using several questionnaires; the first had seven questions to collect socio-demographic information (i.e., age, gender, marital status, education level, diabetes duration, medication regimen, and monthly income). The second was Morisky Medication Adherence Scale (MMAS-8) which is developed by Morisky et al. [21–23]. We used the validated Persian version of the MMAS-8. The internal consistency of the scale was acceptable (Cronbach’s α = 0.697), and test–retest reliability showed excellent reproducibility (r = 0.940, p < 0.001) [24]. Validity was supported by significant correlations with systolic (r = –0.306) and diastolic blood pressure (r = –0.279, both p < 0.001), and by associations between adherence levels and BP control (χ², p = 0.016). The Persian MMAS-8 thus demonstrates acceptable reliability and validity and can be used as a standard tool for assessing medication adherence among Persian-speaking patients. In this study, patients with MMAS-8 scores of <6, 6 to <8, and 8 indicate low, medium, and high adherence, respectively [25]. The third was a HAPA questionnaire, containing 38 questions in eight domains. There were two items about Intention to medication adherence, six items about task self-efficacy of diabetes medication adherence, seven items about Coping self-efficacy, three items about Recovery self-efficacy, two items about Action planning, six items about Coping planning, nine items about Barriers to adherence, and three items about Resources and benefits. The Cronbach’s alpha for the HAPA-based questionnaire exceeded 0.7, and the intraclass correlation coefficient (ICC) was greater than 0.81, indicating good internal consistency and reliability. Test-retest reliability was also confirmed for each scale [26]. Also, we asked an open-ended question about the reasons for non-medication adherence. Given the variation in literacy levels among participants, the method of questionnaire administration was adapted accordingly. For participants with low literacy, trained interviewers conducted face-to-face interviews. During these sessions, each question was read aloud and the participants’ responses were recorded to ensure full understanding and accurate data collection. For literate participants, the questionnaire was self-administered under the supervision of the interviewers, who were available to provide assistance or clarification as needed.

### Analysis

To evaluate the relationships between medication adherence and demographic variables—including age, gender, education level, marital status, and socioeconomic status—we used the chi-square test to identify significant differences. The associations between the dimensions of the Health Action Process Approach (HAPA) and medication adherence were examined using multiple binary logistic regression analysis. This method enabled us to assess the predictive value of each HAPA construct on the likelihood of medication adherence. Descriptive statistics were calculated for all variables. A p-value of less than 0.05 was considered statistically significant. All analyses were conducted using SPSS software, version 13. An open-ended question was posed to participants regarding reasons for non-adherence. The responses were analyzed using conventional content analysis. Two members of the research team (B.KH. and SH.M.) independently reviewed the responses, identified recurring themes, and categorized the reasons for non-adherence based on the frequency of participant reports. Any discrepancies were resolved through discussion, and the most frequently reported categories were summarized.

### Ethics statement

Ethics approval was obtained from the Ethical Review Committee of Ahvaz Jundishapur University of Medical Sciences (Ethics Number: IR.AJUMS.REC.1401.408). This study was conducted under the principles of the Helsinki declaration. Informed consent was obtained from each participant. The funders had no role in study design, data collection, analysis, decision to publish, or preparation of the manuscript.

## Results

### General characteristics of participants

The summary of the participants’ characteristics is shown in Table 1. A total of 179 older adults were enrolled in this study (average age: 64.6 ± 4.99 years; 51.3% women). Furthermore, 81% were married, and 50% of the participants had completed primary education. Most of them (53%) were unemployed. More than 90% of the participants were non-smokers. Eighteen percent of the participants had diabetes for 1–5 years, 22% for 6–10 years, and 67% for 11–20 years. Approximately 24.6% used insulin only, 16.2% used insulin plus oral medications, and 59.2% used oral medications only.

**Table 1:** Baseline patient characteristics.

Variables	Value
Age, years (range 60–89), mean± SD	64.65 ± 4.99
**Gender**	
Female, n (%)	92 (51.4)
Male	87 (48.6)
**Marital status- n (%)**	**146 (81.6)**
single or never married	1
Married	146
Widowed	30
Divorced or separate	2
**Living condition**	
Alone	10
With family	169
**Education level- n (%)**	
No-formal education	50 (27.9)
Elementary School	44 (24.6)
Middle and high School	39 (21.8)
Diploma	26 (14.5)
College level	20 (11.2)
**Occupation – n (%)**	
Unemployed	12 (6.7)
Housewife	83 (46.4)
Employed	28 (15.6)
Retired	56 (33.1)
**Monthly allowance**	
<$559	71 (39.7)
$559 – $783	41 (22.9)
$783 – $1,006	32 (17.9)
> $1,006	35 (19.6)
**Habitat**	
City	165 (92.2)
Rural	14 (7.8)
**Smoking habit**	
Yes	18 (10.1)
No	161 (89.9)
**Diabetes duration (**years**)**	
<1	6 (3.4)
1-5	31 (17.9)
6-10	22 (11.7)
>10	120 (67)
**Type of treatment**	
Insulin only	44 (24.6)
Insulin plus oral medications	29 (16.2)
Oral medications only	106 (59.2)

### Medication adherence levels

Our analysis showed that the percentages of medium and low adherence were 38.8% and 61.2% for oral medication, 38.6% and 61.3% for Insulin only, and 34.5% and 65.5% for insulin plus oral medications, respectively (see Table 2). Based on the results, none of the demographic was a significant predictor of medication adherence (see Table 3).

**Table 2:** Adherence levels by the type of medication.

		Insulin only	Insulin plus medications	oral Medication only	Total
**Morisky score**	**Low, n (%)**	27 (61.3)	19 (65.5)	65 (61.2)	**111 (62)**
	**Medium, n (%)**	17 (38.6)	10 (34.5)	41 (38.8)	**68 (38)**

**Table 3:** Factors influencing medication adherence.

Variables	Low	Medium	Chi-Square	p-value
Participants	111	69		
**Gender- Female, n (%)**	56 (50.5)	26 (52.9)	0.105	0.7
**Marital status**			0.33	0.56
Married	92 (82.9)	54 (79.4)		
Others	19 (17.1)	14 (20.6)		
**Education level**			3.92	0.41
No-formal education	31 (27.9)	19 (27.9)		
Elementary School	27 (24.3)	17 (25)		
Middle and high School	20 (18)	19 (27.9)		
Diploma	18 (16.2)	8 (11.8)		
college level	15 (13.5)	5 (7.4)		
**Occupation**			2.5	0.46
Unemployed	9 (8.1)	3 (4.4)		
Housewife	48 (43.2)	35 (51.5)		
Employed	16 (14.4)	12 (42.9)		
Retired	38 (34.2)	18 (26.5)		
**Monthly allowance**			3.13	0.37
<$559	45 (40.5)	26 (38.2)		
$559 – $783	29 (26.1)	12 (17.6)		
$783 – $1,006	19 (17.1)	13 (19.1)		
> $1,006	18 (16.2)	17 (25)		
**Smoking habit**			2.11	0.14
Yes	14 (12.9)	4 (5.9)		
**Diabetes duration**			25.7	0.1
<1	2	4		
1-5	14	18		
6-10	14	7		
>10	79	41		
**Type of treatment**			0.053	0.8
Insulin	46 (41.4)	27 (39.7)		
Oral medications only	65 (58.6)	41 (60.3)		
Insulin plus oral medications				
**Complication**			0.093	0.76
Yes	86 (61.4)	54 (38.6)		

### Association between medication adherence and the HAPA dimensions

The mean (±SD) scores for all HAPA domains based on the level of medication adherence are reported in Table 4. Logistic regression analysis identified several significant predictors of medication non-adherence. Smoking was positively associated with non-adherence, with smokers being over four times more likely to exhibit low adherence compared to non-smokers (OR = 4.309, 95% CI [1.18, 15.67], *p* = 0.027). Perceived barriers to adherence also significantly increased the likelihood of non-adherence (OR = 1.036, 95% CI [1.01, 1.06], *p* = 0.001). In contrast, higher levels of recovery self-efficacy (OR = 0.924, 95% CI [0.86, 0.99], *p* = 0.027) and coping planning (OR = 0.963, 95% CI [0.93, 0.99], *p* = 0.022) were associated with decreased odds of non-adherence. Other variables, including intention to adhere, perceived resources and benefits, and presence of complications, did not reach statistical significance but showed trends in the expected directions (see Table 5).

**Table 4:** Comparison of HAPA Domain Means Between Medium and Low Medication Adherence.

HAPA domains	Scale Range	Mean (95.0% Confidence Interval)	
		Low medication adherence	Medium medication adherence
Intention to medications adherence	1-7	6.30 (5.96-6.64)	6.84 (6.63–7.05)
Task self-efficacy	1-4	3.61 (3.51-3.71)	3.79 (3.69-3.89)
Coping self-efficacy	1-4	3.80 (3.73-3.86)	3.90 (3.83-3.98)
Recovery self-efficacy	1-4	3.77 (3.68-3.86)	3.98 (3.95-4)
Action planning	1-4	3.84 (3.71-3.93)	3.86 (3.71-4)
Copping planning	1-4	3.49 (3.38-3.60)	3.79 (3.72-3.87)
Barriers to adherence	1-7	3.63 (3.35-3.91)	2.42 (2.18-2.66)
Resources and benefits	1-7	4.27 (3.97-4.56)	4.73 (4.40-5)

**Table 5:** Predictors of Medication Non-Adherence: Results from Logistic Regression.

Model	B	Std. Error	Sig.	Exp(B)	95.0% Confidence Interval for EXP (β)	
					Lower Bound	Upper Bound
Smoking habit	1.461	.693	.027	4.309	1.18	15.67
Recovery self-efficacy	−.079	.036	.027	.924	0.86	0.99
Copping planning	−.038	.016	.022	.963	0.93	0.99
Barriers to adherence	.036	.011	.001	1.036	1.01	1.059
Resources and benefits	−.013	.008	.099	.987	.97	1.002
Intention to medications adherence	−.019	.010	.056	.982	.96	1.001
Complication	−.837	.465	.072	.433	.174	1.077
Constant	13.473	4.123	.001	710148.620		

### Reasons of medication non-adherence

The participants’ reasons for suboptimal medication adherence are classified into 12 categories. The three top common self-reported reasons for suboptimal medication adherence were lack of affordability (17.5%), lack of family support (10%), and poor understanding of disease (9.4%) (See Table 6).

**Table 6:** Barriers to medication adherence.

Factors	Frequency	Percent
Costs of drugs and treatment (co-payment)	28	17.5%
Lack of family support	16	10.0%
Poor understanding of disease (low health literacy)	15	9.4%
Clinical improvement, disappearance of symptoms, feeling better/worse	14	8.8%
physical problems, depression	14	8.8%
Forgetting	13	8.1%
Inadequate instruction or counseling	13	8.1%
Duration of therapy	12	7.5%
Difficulty of time schedule, complexity of the regimen, dosing frequency	12	7.5%
Disbelief in medicine effectiveness	10	6.3%
Adverse effects	6	3.8%
Other reasons	7	4.4%

## Discussion

This study investigated medication adherence levels and their predictors in older patients with type 2 diabetes, assessing adherence based on the Health Action Process Approach (HAPA). Our findings reveal that a majority of participants exhibited suboptimal medication adherence. Lack of affordability, lack of family support, and poor understanding of disease were the most common identified reasons for poor adherence to medication. Utilizing the HAPA framework, we identified several cognitive factors predictive of medication adherence. Specifically, recovery self-efficacy, coping planning, and perceived barriers emerged as significant predictors of medication adherence behavior among patients.

Our results indicating suboptimal medication adherence align with findings from previous research. A review of 27 studies reported adherence rates ranging widely from 38.5% to 93.1%, with only 22.2% of the studies observing adherence rates of 80% or higher [13]. In Jordan, 7.9% of elderly patients demonstrated low adherence, 33.6% moderate adherence, and 58.5% high adherence to their prescribed medications [27]. Similarly, studies conducted in Iran reported poor adherence rates of 72.6% [28]and 47.89% [29] among patients, respectively. Taken together, these findings underscore the widespread issue of suboptimal adherence among elderly patients with type 2 diabetes, consistent with trends observed across various low- and middle-income countries.

Our results align with previous research emphasizing the global scope of suboptimal adherence to diabetes medication [1,13]. However, we found no significant correlation between medication adherence and patients’ characteristics. In contrast, Serap et al. found a significant relationship between education level, diabetes duration, and medication non-adherence [30]. Similarly, Kirkman et al. reported that younger patients, those newly diagnosed with diabetes, and those taking fewer medications may be at higher risk of nonadherence [31]. Additionally, Waari et al. demonstrated significant associations between patient age, gender, and medication adherence [32]. while Elsous et al. reported that females with longer durations of diabetes mellitus were more likely to adhere to anti-diabetic medications [33]. One possible explanation is the relative homogeneity of our sample, consisting of older adults, most of whom lived with family members and had low to medium education levels. This homogeneity may have attenuated the influence of variables such as education, marital status, or income. Furthermore, discrepancies across study results may be attributed to cultural differences and variations in participant characteristics. In addition, we did not examine contextual factors such as healthcare access, medication costs, and family support systems, which may play a stronger role in shaping adherence than demographic characteristics.

Based on our finding, the identified perceived barriers—such as difficulty in timely medication or insulin injection, pain, cost concerns, fear of stigma, and forgetfulness—can significantly hinder patients’ adherence to prescribed treatments. Understanding these barriers is crucial for designing effective interventions that address patients’ specific challenges.

Self-efficacy is another predictor of behavior in our study. In terms of self-efficacy, people have confidence in their ability to change [34] and to overcome obstacles in changing behaviors. However, individuals with high self-efficacy believe in their capability to effectively follow medical recommendations [35]. Therefore, it is essential to provide the required information to patients and involve them in their care [36]. Related factors to low medication adherence are patient-centered (e.g., poor health literacy, lack of confidence in health care providers and lack of participation in the treatment process), physician-centered (e.g., prescription of complex medication regimens and ineffective communication), and health care systems (e.g., limited access to care, and lack of health information technology, lack of integrated care) [37,38]. To build self-efficacy, involving patients in shared decision-making can foster a sense of control and competence. Strengthening this belief should be a central focus in adherence interventions.

Coping planning is another identified dimension that suggests people should be able to imagine scenarios in which they are unable to engage in their intended behavior, or they should form plans to manage any barriers to medication adherence. Coping planning significantly predicts medication adherence behavior. Like our results, Trevisan et al. found that coping planning as a behavioral strategy helped improve medication adherence [39]. Several strategies—such as planning for temptations to skip doses, managing disruptions, using reminders, and knowing how to self-inject or seek help—can be helpful. Incorporating these coping strategies into interventions may effectively bridge the intention–behavior gap and support adherence.

Although intention to adhere to medications was not a statistically significant predictor in our model, the direction of the effect suggested that stronger intentions were associated with lower odds of non-adherence. Intentions define a motivation regarding a goal or target behavior. However, without explicit intentions, changes in habitual behavior patterns are unlikely to occur [40]. However, similar to our findings, McCleary et al. reported that while most participants expressed strong intentions to adhere to their medication, actual adherence rates remained sub-optimal [41]. This suggests that intention alone may not be sufficient to ensure adherence, and that additional self-regulatory factors, such as coping planning and self-efficacy, are necessary to translate intention into sustained behavior.

The top reasons for suboptimal medication adherence among participants were high co-payment, a lack of support and empathy from family, and poor understanding of disease, feeling better or worse, patients’ physical disabilities, forgetting to take medication, feeling uncomfortable taking insulin. Cesar found that patients with low disease severity thought they did not need medications [42]. This finding is partly consistent with our results, as both highlight patients’ perceptions and beliefs about their illness and treatment as important factors influencing adherence. Low health literacy about diabetes self-management practices, cultural practices related to diabetes self-management, lack of availability and accessibility of resources, and financial problems were identified as the most important barriers to medication non-adherence based on the literature [43,44]. Collectively, these findings point to the fact that adherence is shaped not only by individual behaviors but also by social, cultural, and structural determinants. Addressing these barriers therefore requires a multi-level approach: policymakers could reduce financial strain by subsidizing medications for older adults, while healthcare providers might improve outcomes by involving family members in counselling, tailoring education to patients’ cultural contexts, and enhancing support for those with physical or cognitive limitations.

This study has several limitations. First, self-reported adherence data may be affected by social desirability and recall biases, potentially impacting accuracy. Second, the study was limited to two public clinics, which may restrict generalizability to other settings. Third, the cross-sectional design precludes causal inferences, allowing only for the identification of associations.

## Conclusions

This study highlights that older people had suboptimal adherence to medication. Smoking and perceived barriers were significant risk factors, increasing the likelihood of poor adherence. Conversely, higher levels of recovery self-efficacy and coping planning served as protective factors, reducing the risk of non-adherence. Policymakers and planners should consider the mentioned factors in designing interventions to change behavior for chronic diseases like diabetes.
