# Expression of Concern: Determinants of medication adherence among older adults with type 2 diabetes using the health action process approach: A cross-sectional study

**DOI:** 10.1371/journal.pone.0357069

**Published:** 2026-08-27

**Authors:** The PLOS One Editors

After this article [1] was published, concerns were noted regarding its use of the Morisky Medication Adherence Scale-8 (MMAS-8), published in [2], which was retracted due to concerns regarding the reported specificity and sensitivity values [3].

The corresponding author stated that they used the validated Persian version of MMAS-8 [4], cited in [1] as Reference 24, which independently reports sensitivity and specificity values. They further stated that [1] does not assess or rely upon the sensitivity, specificity, predictive validity, or screening accuracy reported in [2].

The *PLOS One* Editors issue this Expression of Concern to inform readers of the above issue and to advise that findings in [1] based on MMAS-8 adherence classifications should be interpreted with caution.
